# Kinetic Analysis by Affinity Chromatography

**DOI:** 10.3389/fchem.2019.00673

**Published:** 2019-10-18

**Authors:** Sazia Iftekhar, Susan T. Ovbude, David S. Hage

**Affiliations:** Department of Chemistry, University of Nebraska-Lincoln, Lincoln, NE, United States

**Keywords:** affinity chromatography, biological interactions, kinetics, peak profiling, peak decay method, plate height method, split-peak method, ultrafast affinity extraction

## Abstract

Important information on chemical processes in living systems can be obtained by the rates at which these biological interactions occur. This review will discuss several techniques based on traditional and high-performance affinity chromatography that may be used to examine the kinetics of biological reactions. These methods include band-broadening measurements, techniques for peak fitting, split-peak analysis, peak decay studies, and ultrafast affinity extraction. The general principles and theory of each method, as applied to the determination of rate constants, will be discussed. The applications of each approach, along with its advantages and limitations, will also be considered.

## Introduction

The analysis of biological interactions is important in describing and studying the processes that occur in living systems. Many biological interactions are due to non-covalent processes that involve drugs, hormones, proteins, peptides, metal ions, nucleic acids, and lipids (Myszka and Rich, 2000; Schreiber et al., 2009; Vuignier et al., 2010; Williams, 2013; Zheng et al., 2014a, 2015a; Bi et al., 2015). For instance, transport proteins such as α_1_-acid glycoprotein (AGP) and human serum albumin (HSA) can bind to and carry many drugs within the circulatory system through non-covalent interactions, thereby affecting the absorption, distribution, metabolism, and excretion of these drugs in humans (Zheng et al., 2014a; Bi et al., 2015). Information on the kinetics of a biological interaction can help determine the function of these interactions, as well as the mechanisms through which they occur (Myszka and Rich, 2000; Schreiber et al., 2009; Vuignier et al., 2010; Williams, 2013; Zheng et al., 2014a, 2015b; Bi et al., 2015).

There are a variety of techniques that can be used to study the kinetics of a biological system and to measure the rate constants for this type of process. These methods are currently chosen based on the type of the system that is under investigation, the complexity of the reaction, the rates of the corresponding reactions, and the amounts and concentrations of the reactants and products that are required and available. Approaches that have been used in the past to study reaction rates in biological systems are surface plasmon resonance spectroscopy (SPR), capillary electrophoresis (CE), and stopped-flow analysis (Myszka and Rich, 2000; Krylov, 2007; Williams, 2013; Zheng et al., 2015b). However, each of these methods possesses several disadvantages. For instance, there is the need for a sufficient concentration of the reactant or product and a measurable signal in stopped-flow analysis, the requirement of a special type of surface in SPR, and the need for a measurable difference in mobilities between the products and reactants in CE. There is also the need to consider adsorption of biomolecules to the capillary surface in CE, which can be minimized through capillary treatment with a suitable polymer (Zheng et al., 2015b).

High-performance affinity chromatography (HPAC) is an alternative tool that can be used to determine rate constants in biological systems. HPAC is a form of affinity chromatography which uses a biologically-related binding agent as a stationary phase, which is then placed in a column suitable for high-performance liquid chromatography (HPLC). This binding agent, or affinity ligand, may consist of an immobilized protein, enzyme or antibody; enzyme substrate or inhibitor; antigen; biomimetic dye; and DNA or RNA sequence, among others (Hage, 2006; Schiel and Hage, 2009). HPAC makes use of small and rigid supports such as silica or monoliths which can be used with the immobilized agent to provide rapid and efficient separations. In both HPAC and traditional affinity chromatography, the target analyte undergoes selective and reversible interactions with the binding agent, which allows the target to be captured and retained as it goes through the column (Hage et al., 2012; Bi et al., 2015; Zheng et al., 2015b; Zhang et al., 2018). HPAC and affinity chromatography are often used as separation methods to purify or analyze a given compound or group of related solutes in an applied sample. This has included use of these methods for sample pretreatment, flow-based immunoassays, chiral separations, and multi-dimensional methods (Hage, 2006). However, HPAC and related affinity methods have also been employed to characterize the strength, binding sites, and rates of biological interactions. Examples of systems that have been investigated with such an approach include binding by drugs with serum proteins, antibody-antigen interactions, binding of enzymes with substrates or inhibitors, interactions of immunoglobulin-binding proteins with antibodies, and binding of glycoproteins by lectins (Hage, 2006; Schiel and Hage, 2009; Zhang et al., 2018).

**Figure 1:**
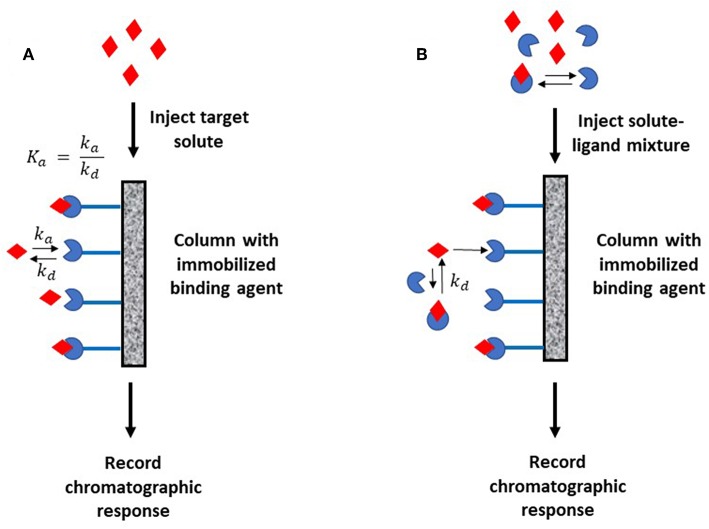
Two general formats employed in affinity chromatography and HPAC for examining the kinetics of an analyte interaction with a binding agent. The method in **(A)** is based on the reversible interactions between the analyte and an immobilized binding agent, while **(B)** is based on the utilization of a secondary probe on the column to capture part of the analyte and monitor interactions of this analyte with a binding agent in solution. Terms: *K*_*a*_, association equilibrium constant; *k*_*a*_, association rate constant; *k*_*d*_, dissociation rate constant.

Figure 1 shows two general formats that have been employed in HPAC and affinity chromatography for determining rate constants. In Figure 1A, the target analyte is introduced into an affinity column containing the binding agent in an immobilized form. In this format, the retention and/or elution behavior of the analyte provides information on the dissociation or association rates between the target analyte and binding agent (Schiel and Hage, 2009). The second format is shown in Figure 1B. In this approach, the analyte is introduced into the column along with a soluble binding agent. An immobilized binding agent in the column acts as a secondary probe to examine the analyte-binding agent interactions in solution by capturing the target in its free, non-bound state (Bi et al., 2015; Zheng et al., 2015b).

The choice of a method for characterizing the rates of analyte-ligand binding by HPAC and affinity chromatography will depend on several factors. Some important considerations are the overall binding strength and rate of the interaction that is to be observed and the type of information that is required (Schiel and Hage, 2009; Zheng et al., 2014a, 2015b; Bi et al., 2015). Other considerations are the amounts of analyte and binding agent that are available for analysis. For instance, some affinity-based methods rely on application of a target analyte in a continuous manner (i.e., a format known as frontal affinity chromatography or frontal analysis), while others require injection of only a small quantity of analyte (i.e., an approach known as zonal elution). Methods in which the analyte may approach the amount of binding agent often involve the use of non-linear elution conditions, in which the chromatographic response will depend on the amount of applied analyte. Techniques that need only small amounts of analyte may instead use or require linear elution conditions, in which the observed chromatographic behavior is independent of the amount of analyte (Schiel and Hage, 2009).

Proper selection of the method for immobilizing a binding agent is another important factor to consider in affinity chromatography (Hage and Kim, 2006; Schiel et al., 2006). This is particularly true when the immobilized agent is used directly for binding or kinetic studies. A good coupling method should result in an immobilized agent that is properly oriented and that has high activity, good stability, and easy accessibility to its targets (Hage and Kim, 2006). Many applications of affinity chromatography have used covalent coupling techniques that employ an activated support and amine, hydroxyl, carbonyl or sulfhydryl groups on the binding agent; this approach has been used for a number of proteins and other biological agents (Hage and Kim, 2006; Schiel et al., 2006; Beeram et al., 2018; Liang et al., 2018). Smaller targets can be attached through similar groups to an activated support and by using a spacer arm to avoid steric hindrance effects during binding (Hinze et al., 1985; Wang et al., 2014, 2016). Sometimes adsorption can be used for immobilization. For instance, adsorption has been utilized with membrane-associated receptor proteins that have been placed within immobilized artificial membrane columns (Moaddel et al., 2005). In all of these methods the immobilization technique should be chosen to produce a binding agent that is a good mimic of the same binding agent in its native state. The extent to which this is achieved should be checked and validated by examining interactions of the immobilized agent with model targets that have known binding properties; this should be done prior to further studies with the same binding agent and new or unknown targets (Schiel and Hage, 2009; Bi et al., 2015; Zheng et al., 2015b).

This review will examine several techniques that can be utilized in HPAC or affinity chromatography for studying the kinetics of biologically-relevant systems. A short summary of this topic was provided recently (Bi et al., 2015) and will be expanded upon in this paper, with greater emphasis on the basic principles, theory, and use of HPAC and affinity methods for kinetic studies. The approaches that will be considered are band-broadening measurements, peak decay analysis, the split-peak method, techniques for peak fitting, and ultrafast affinity extraction. Applications of these methods will be discussed, and the advantages and potential limitations of each technique will be considered.

## Band-Broadening Measurements

An analysis of band-broadening in affinity chromatography is one way kinetic information can be obtained on the interaction of an analyte with a given binding agent (Schiel and Hage, 2009; Bi et al., 2015; Zheng et al., 2015b). This type of method is based on the injection of the target analyte onto both a column containing the binding agent and an inert control column. These injections are typically made under linear elution conditions, with the data from the control column being used to correct for the contributions due to band-broadening processes besides stationary phase mass transfer. The measured peak widths are then used to calculate the plate heights of the system and may also be used to make a van Deemter-type plot in which the total plate height is analyzed as the linear velocity, or flow rate, is varied. This plate height data is then used to provide information on the broadening of peaks that results from stationary phase mass transfer, which is related to the interaction rate between the immobilized binding agent and target. Band-broadening measurements for kinetic studies can be carried out by using two related methods: the plate height method and peak profiling (Schiel and Hage, 2009; Zheng et al., 2014a, 2015b; Bi et al., 2015). Details on each method are provided in the following sections.

### Plate Height Method

**Figure 2:**
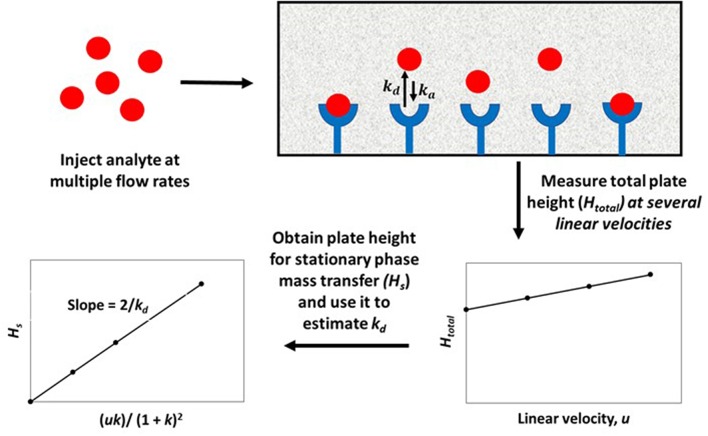
General scheme for studying an analyte-binding agent interaction by using the plate height method. Terms: *H*_*total*_, total plate height; *H*_*s*_, plate height contribution due to stationary phase mass transfer; *u*, linear velocity of mobile phase; *k*, retention factor of the analyte; *k*_*d*_, dissociation rate constant.

The plate height method for kinetic studies makes use of a small quantity of a target that is injected onto an affinity column, as well as onto a control column, at several flow rates. The band-broadening data that are collected from these columns are then used to acquire the total plate height, *H*_*total*_, on each column, as well as to estimate and compare the plate height contributions due to individual band-broadening processes on these columns. The resulting information can be combined to find the stationary phase mass transfer plate height contribution, *H*_*s*_, for the affinity column as a function of the mobile phase's linear velocity (*u*) and the target's retention factor (*k*) for the affinity column. This process is illustrated in Figure 2 (Schiel and Hage, 2009; Bi et al., 2015; Zheng et al., 2015b).

In describing the band-broadening processes that are present in the columns, the term *H*_*s*_ is related to the rate of analyte dissociation from the immobilized binding agent. This relationship is shown in Equation (1) (Schiel and Hage, 2009; Bi et al., 2015; Zheng et al., 2015b).

(1) $$\begin{array}{l} H_{s}\;=\;\frac{2uk\;}{{\left(1+k\right)}^{2}k_{d}} \end{array}$$

**Figure 3:**
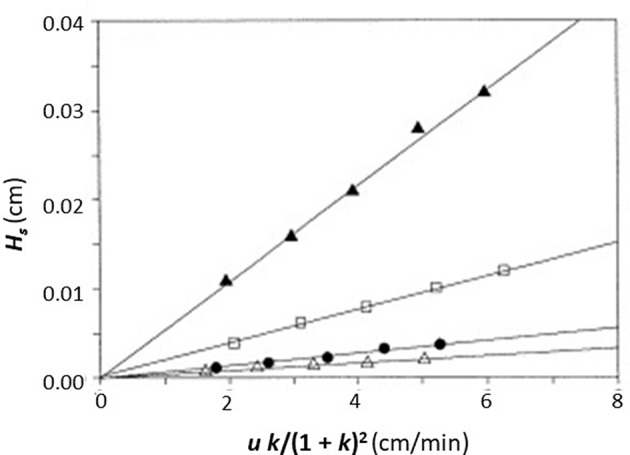
Plot of *H*_*s*_ vs. (*uk*)/(1 + *k*)^2^, as obtained by the plate height method, to analyze the binding of D-tryptophan with an HPAC column containing immobilized HSA. These data were obtained at 25°C and at mobile phase pH values of 4.0 (Δ), 5.0 (•), 6.0 (□), or 7.0 (▲). Reproduced with permission from Yang and Hage (1997). Copyright 1997 Elsevier.

In this equation, the dissociation rate constant for the target analyte from the immobilized binding agent is given by the term *k*_*d*_. This equation assumes that the analyte's interactions within the column are occurring with a single set of binding sites and that the injected amount of analyte is small compared to the amount of binding agent that is active and present in the column (i.e., it is assumed the experiment is done under linear elution conditions). When a plot is made of *H*_*s*_ vs. (*uk*)/(*1*+*k*)^2^ based on Equation (1), a system which follows these assumptions should provide a linear best-fit response with a slope that has a value of 2/*k*_*d*_ and an intercept that is equal to zero. Figure 3 shows an example of a plot for *H*_*s*_ vs. (*uk*)/(*1* + *k*)^2^ that was obtained by HPAC when this method was used to examine the dissociation kinetics for D-tryptophan in the presence of immobilized HSA (Yang and Hage, 1997).

Once the value of *k*_*d*_ has been obtained, the association rate constant (*k*_*a*_) for an analyte with an immobilized binding agent can also be acquired. This can be found by using the measured value of *k*_*d*_ with a separate known or measured equilibrium constant for the same system. For instance, if the association equilibrium constant (*K*_*a*_) for the particular interaction is determined by an approach such as frontal analysis, *k*_*a*_ can be estimated by using Equation (2) (Schiel and Hage, 2009; Bi et al., 2015; Zheng et al., 2015b).

(2) $$\begin{array}{l} k_{a}\;=K_{a}k_{d} \end{array}$$

The plate height technique has been utilized to study the interactions of chiral solutes such as D/L-tryptophan and *R/S*-warfarin with HSA, as well as the effect of temperature on the rates of these interactions (see summary in Tables 1, 2 of applications for all methods discussed in this review) (Loun and Hage, 1996; Yang and Hage, 1997). Early work with this method used it to examine the interaction rates of various sugars with the lectin concanavalin A (Con A) (Anderson and Walters, 1986). This approach has also been employed to investigate the effects of pH, solvent polarity, and ionic strength with regards to the interaction rates of D- and L-tryptophan with HSA (Yang and Hage, 1997). In addition, this method has been used to assess the employment of monoliths and small affinity columns for studying the interaction kinetics of HSA with *R*-warfarin, L-tryptophan, and carbamazepine (Yoo and Hage, 2009; Yoo et al., 2010).

**Table 1:** General range of rate constants that have been determined by methods using affinity chromatography and HPAC^a^.

**Analysis method**	**Conditions**	**Range of binding affinities (*K_***a***_*)**	**Range of measured rate constants**
Plate height method	Linear elution conditions; zonal elution format	10^3^-10^6^ M^−1^	*k_*d*_*: 10^−2^-10 s^−1^
Peak profiling	Linear elution conditions; zonal elution format	10^3^-10^6^ M^−1^	*k_*d*_*: 10^−1^-10 s^−1^
Peak decay method	Non-linear elution conditions; zonal elution format	10^3^-10^6^ M^−1^	*k_*d*_*: 10^−2^-10 s^−1^
Split-peak method	Non-linear or linear elution conditions; zonal elution format	>10^6^ M^−1^	*k_*d*_*: 10^−1^ s^−1^ *k_*a*_*: 10^4^-10^6^ M^−1^ s^−1^
Peak fitting	Non-linear elution conditions; zonal elution or frontal analysis format	10^3^-10^6^ M^−1^	*k_*d*_*: 10^−1^-10 s^−1^*k_*a*_*: 10^4^-10^7^ M^−1^ s^−1^
Ultrafast affinity extraction	Solution-phase interactions; zonal elution format	10^3^-10^9^ M^−1^	*k_*d*_*: 10^−2^-10 s^−1^*k_*a*_*: 10^3^-10^5^ M^−1^ s^−1^

a *Terms: K_a_, association equilibrium constant; k_a_, association rate constant; k_d_, dissociation rate constant*.

**Table 2:** Applications of affinity chromatography and HPAC in kinetic studies^a^.

**Technique**	**System examined**
Plate height method	Drug and solute binding with serum proteins (HSA); sugar-lectin interactions (Con A)
Peak profiling	Drug, drug metabolite, and solute binding with serum proteins (AGP, HSA); drug binding with cyclodextrins; drug-receptor interactions (β_2_-AR)
Peak decay	Drug binding with serum proteins (AGP, HSA); sugar-lectin interactions (Con A); antibody-antigen binding; aptamer-target interactions; interactions of immunoglobulins with protein A or protein G
Split-peak method	Antibody-antigen binding; interactions of immunoglobulins with protein A or protein G
Peak fitting in zonal elution	Sugar-lectin interactions (Con A); drug/inhibitor-receptor interactions (nAChR, β_2_-AR); drug binding with cyclodextrins; interactions of immunoglobulins with protein A; binding of novobiocin with heat shock protein; lysozyme binding with Cibacron Blue 3GA
Peak fitting in frontal analysis	Sugar-lectin interactions (Con A); antibody-antigen binding
Ultrafast affinity extraction	Drug and hormone binding with serum proteins (AGP, HSA, SHBG);

a *AGP, α_1_-acid glycoprotein; β_2_-AR, beta_2_-adrenoceptor; Con A, concanavalin A; HSA, human serum albumin; nAChR, nicotinic acetylcholine receptor; SHBG, sex-hormone binding globulin*.

The plate height method is suitable for studying reactions that have relatively fast association and dissociation rates compared to the time needed for elution from the affinity column. The range of dissociation rate constants that have been determined by this method is ~10^−2^-10^−1^ s^−1^ (see summary in Table 1) (Loun and Hage, 1996; Schiel and Hage, 2009; Yoo et al., 2010; Bi et al., 2015; Zheng et al., 2015b). Systems which exhibit weak-to-moderate binding (i.e., *K*_*a*_ ≤ 10^6^ M^−1^) have been typically studied using this method (Loun and Hage, 1996; Yang and Hage, 1997; Schiel and Hage, 2009; Yoo and Hage, 2009; Yoo et al., 2010; Zheng et al., 2015b). An advantage of this approach is only a small amount of the analyte is needed, as is required to achieve linear elution conditions. A potential limitation of this method is that a detailed analysis of the affinity and control columns, which may include the use of a large number of flow rates and many replicate injections, may be needed to obtain sufficiently precise values for contributions to the plate height by various processes (Schiel and Hage, 2009; Bi et al., 2015; Zheng et al., 2015b).

### Peak Profiling

Peak profiling is a variation of the plate height technique that typically requires work at fewer flow rates and involves more direct calculations of dissociation rate constants (Fitos et al., 2002; Talbert et al., 2002; Schiel et al., 2009; Tong et al., 2011). This technique is based on the measurement of both the retention time and peak variance (i.e., band-broadening) of an analyte on a control column and an affinity column under linear elution conditions. This approach can ideally be carried out at a single flow rate if it is assumed all band-broadening sources besides stationary phase mass transfer are not significant or the same for the analyte in both the affinity column and control column. The apparent dissociation rate constant (*k*_*d, app*_) in this situation can be calculated by using the measured parameters along with Equation (3) (Schiel and Hage, 2009).

(3) $$\begin{array}{l} k_{d,app\;}=\;\frac{2t_{M\;}^{2}\left(t_{R\;-\;}t_{M}\;\right)\;}{\sigma_{R}^{2}t_{M}^{2}\;-\;\sigma_{M}^{2}t_{R}^{2}} \end{array}$$

In this equation, the retention or elution times of the analyte on the affinity column and control column are given by *t*_*R*_ and *t*_*M*_, respectively. The terms $\sigma_{R}^{2}$ and $\sigma_{M}^{2}$ represent the variances of the peaks for the same analyte on the affinity column and control column (Schiel and Hage, 2009).

**Figure 4:**
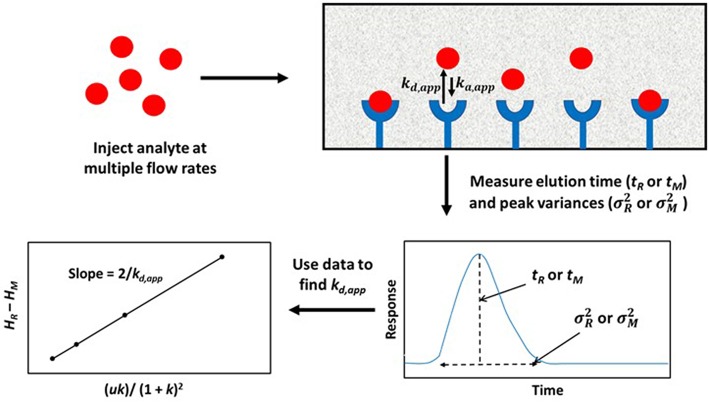
General scheme used for studying analyte interactions with a binding agent by using peak profiling. Terms: *t*_*R*_, retention time of the analyte on an affinity column; *t*_*M*_, elution time of a non-retained solute or analyte on a control column; $\sigma_{R}^{2}$, peak variance of analyte on the affinity column; $\sigma_{M}^{2}$, peak variance of the non-retained solute or analyte on a control column; *H*_*R*_, total plate height obtained for analyte on the affinity column; *H*_*M*_, total plate height obtained for non-retained solute or analyte on a control column; *u*, linear velocity of mobile phase; *k*, retention factor of analyte on the affinity column; *k*_*d,app*_, apparent dissociation rate constant; *k*_*a*_,_app_, apparent association rate constant.

A modified form of the peak profiling method looks at the difference in total plate heights that are found under linear elution conditions for the analyte on an affinity column (*H*_*R*_) and a control column (*H*_*M*_). A plot of this difference, *H*_*R*_–*H*_*M*_, is then made vs. the term (*uk*)/(*1*+*k*)^2^. An example of such a plot is provided in Figure 4. Equation (4) can then be employed with the data to obtain *k*_*d,app*_ (Schiel and Hage, 2009; Schiel et al., 2009; Bi et al., 2015; Zheng et al., 2015b).

(4) $$\begin{array}{l} H_{R}-H_{M}\;=\frac{2uk}{{\left(1+k\right)}^{2}k_{d,app}\;}\;=\;H_{s} \end{array}$$

**Figure 5:**
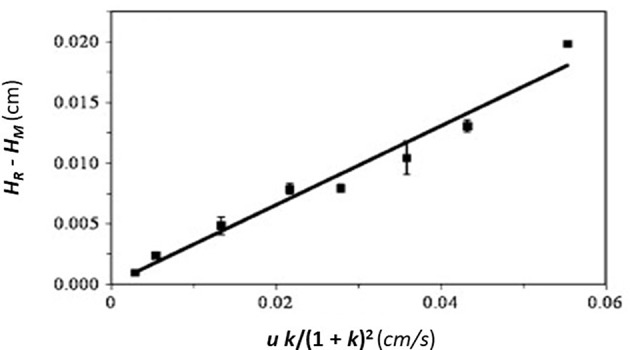
Plot of *H*_*R*_–*H*_*M*_ vs. (*uk*)/(1 + *k*)^2^, as obtained by peak profiling for the injection of 5-(4-hydroxyphenyl)-5-phenylhydantoin (*p*-HPPH) onto a column containing immobilized HSA. Reproduced with permission from Tong and Hage (2011). Copyright 2011 Elsevier.

This equation can be used with data that have been obtained for the injection of an analyte at either single or multiple flow rates. This particular expression indicates the behavior that is expected for an analyte at a single type of binding site on the affinity column. If data are acquired at multiple flow rates, a plot of *H*_*R*_–*H*_*M*_ vs. (*uk*)/(1 + *k*)^2^ should provide a linear relationship in which the slope is equal to the term 2/*k*_*d,app*_(Schiel et al., 2009; Tong et al., 2011). Figure 5 provides an example of a plot that has been obtained by this method (Tong and Hage, 2011).

Peak profiling can also be used to determine dissociation rate constants for systems that have multiple types of binding sites for the analyte within a column. This can be accomplished by using Equation (5) or related expressions.

(5) $$\begin{array}{l} H_{R}-H_{M}\;=\;\frac{uk}{{\left(1+k\right)}^{2}\;}\;\left[\frac{2\alpha_{1}\;}{k_{d}\;}+\frac{2\;\alpha_{control}\;}{k_{d,control}}\right] \end{array}$$

In this equation, the terms α_1_ and α_*control*_ represent the fractions of the total retention factor for the target that are due to interactions with the immobilized binding agent or due to non-specific binding to the support (i.e., as estimated using a control column). The term *k*_*d,control*_ is the dissociation rate constant for the retained target as it interacts with the non-specific binding sites (Tong and Hage, 2011; Tong et al., 2011).

An expanded form of Equation (4) can be used in cases where a correction must be made for the change in mass transfer due to the stagnant mobile phase as the degree of analyte is varied. This revised form is given by Equation (6) (Schiel and Hage, 2009; Schiel et al., 2009).

(6) $$\begin{array}{l} H_{R}-H_{M}\;=\;\frac{uk}{{\left(1+k\right)}^{2}\;}\;\left[\frac{2}{k_{d}}+\;\frac{d_{p}^{2}\;\left(2+3k\right)}{60\;\gamma D}\right] \end{array}$$

The term *d*_*p*_ in this equation is the particle diameter of the support, γ is the tortuosity factor for analyte movement in this support, and *D* is the analyte's diffusion coefficient in the mobile phase. Based on this expression, a plot of *H*_*R*_*-H*_*M*_ vs. [*uk*/(1 + *k*)^2^] can be made for columns that contain supports with various, known particle diameters. A linear plot should be obtained for systems with single-site binding. The slopes of these plots can then be plotted against $d_{p}^{2}$ to obtain a new graph in which the true value of *k*_*d*_ is obtained from the intercept (Schiel and Hage, 2009; Schiel et al., 2009).

The peak profiling technique has been used to characterize a number of systems. For instance, this approach has been utilized to study the dissociation kinetics of drugs/solutes such as imipramine, carbamazepine and L-tryptophan with immobilized HSA (Schiel and Hage, 2009; Tong et al., 2011); the interactions of acetaminophen and sertraline with β-cyclodextrin (Li et al., 2013); and the interactions of beta_2_-adrenoceptor (β_2_-AR) with drugs such as salbutamol, terbutaline, methoxyphenamine, isoprenaline hydrochloride, and ephedrine hydrochloride (Liang et al., 2018). The same general method has been applied to characterizing dissociation rate constants for AGP with the drugs chlorpromazine, disopyramide, imipramine, lidocaine, propranolol, and verapamil based on affinity microcolumns (Beeram et al., 2017). A simultaneous determination of the dissociation rate constants for two chiral phenytoin metabolites with HSA was reported with this method (Tong and Hage, 2011), and a modified peak profiling method based on multianalyte detection has been employed to study interactions of β-cyclodextrin with drugs such as acetaminophen, phenacetin and *S*-flurbiprofen (Wang et al., 2014). Peak profiling has been used in most of these studies with absorbance detection; however, it has also been used with tandem mass spectrometry to characterize the binding kinetics of acetaminophen, trimethoprim, ketoprofen, indapamide, and uracil with β-cyclodextrin (Wang et al., 2016).

The advantages and potential limitations of peak profiling are similar to those already discussed for the plate height method. The dissociation rate constants obtained by this method have been in the range of 10^−1^-10^1^ s^−1^, and this approach has generally been used with systems that have weak-to-moderate binding strengths (*K*_*a*_ ≤ 10^6^ M^−1^) (Schiel et al., 2009; Tong and Hage, 2011; Tong et al., 2011; Li et al., 2013; Wang et al., 2014, 2016; Beeram et al., 2017; Liang et al., 2018). Some advantages of this approach over the plate height method are that it can be employed at one or many flow rates and it can use higher flow rates than the plate height method, resulting in higher throughput measurements and faster data collection (Schiel and Hage, 2009; Bi et al., 2015; Zheng et al., 2015b).

## Peak Decay Method

In peak decay methods, a small plug of a target solute is applied to both a small affinity column and control column, followed by release of the retained analyte under conditions where it does not have an opportunity to rebind. These latter conditions can be achieved by using relatively high flow rates and selective mobile phase conditions to prevent rebinding by the target to the immobilized binding agent and to avoid movement of the analyte back into the stagnant mobile phase. This technique can be performed in two formats: the competitive peak decay method and the non-competitive peak decay method (Schiel and Hage, 2009; Bi et al., 2015; Zheng et al., 2015b).

**Figure 6:**
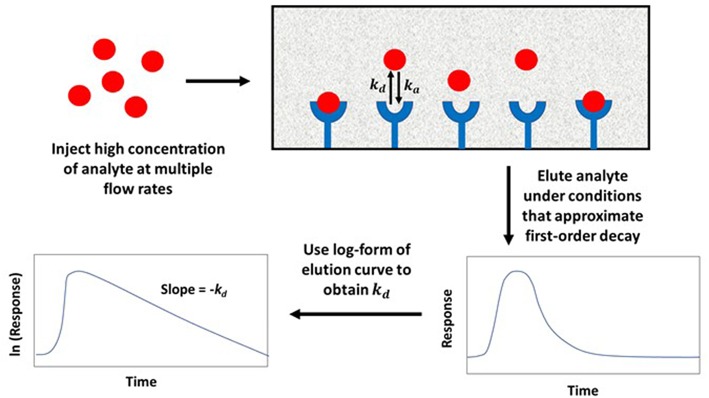
General scheme used for studying analyte interactions with an immobilized binding agent by the non-competitive peak decay method. Terms: *k*_*d*_, dissociation rate constant; *k*_*a*_, association rate constant.

In the non-competitive method, a significantly large amount of analyte is applied to both a small affinity column and a control column at various flow rates. This causes the target solute to interact and partially saturate the column. Once the analyte is released from the column, any non-bound target in the stagnant mobile phase is washed away, along with target that is non-specifically and weakly-retained by the support. The bound target is then washed from the column at a relatively high flow rate as it dissociates from the immobilized agent. The decay curve or elution profile that is produced is then monitored, as illustrated in Figure 6 (Chen et al., 2009; Schiel and Hage, 2009; Bi et al., 2015; Zheng et al., 2015b).

The competitive peak decay method has the analyte being introduced into an affinity column and eluted by adding a high concentration of a competing agent. This competing agent binds to the same site of the immobilized agent as the target and prevents re-association of the target as it is released from the column. This produces a scenario in which the target analyte is continuously washed from the affinity column as it is dissociated from the binding agent, creating a decay profile for elution. One disadvantage of this method is that it cannot be used for systems in which both the competing agent and target exhibit a significant response, making it difficult to monitor the target's elution (Schiel and Hage, 2009; Bi et al., 2015; Zheng et al., 2015b).

The elution profile in both of these methods approaches a first-order decay curve when the target analyte is completely prevented from rebinding to the column as it is released from the immobilized binding agent. If dissociation of the analyte is slower than mobile phase mass transfer, the logarithm of the response for the tailing portion of the resulting peak can be plotted against time and used to obtain *k*_*d*_. This can be done by employing Equation (7) (Schiel and Hage, 2009; Bi et al., 2015; Zheng et al., 2015b).

(7) $$\begin{array}{l} \text{ln}\left(\frac{dm_{Ee}\;}{dt}\right)=\;\ln\left(m_{Eo}k_{d}\right)-\;k_{d}t \end{array}$$

**Figure 7:**
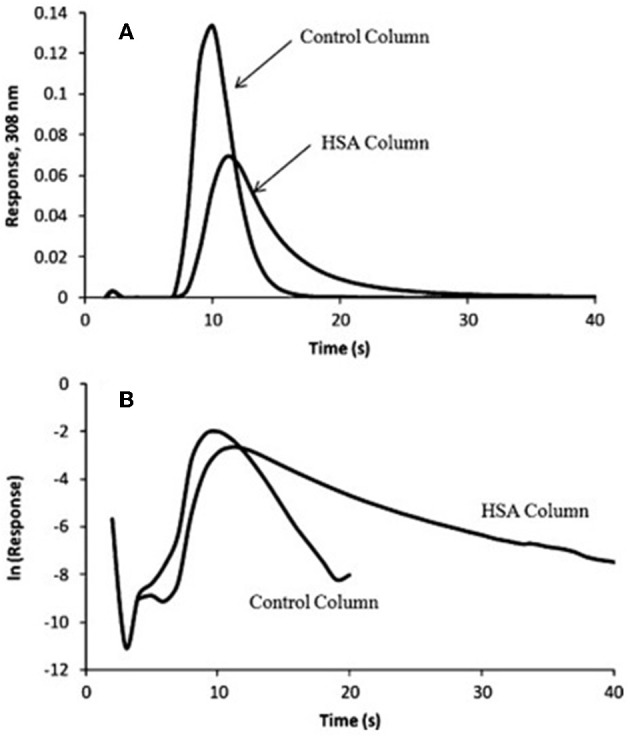
**(A)** Elution profile and **(B)** natural logarithm of the elution profile obtained for the application of a 100 μL sample of 10 μM racemic warfarin onto a control monolith column and an HSA monolith column and by using the peak decay method for data analysis. Reproduced with permission from Yoo and Hage (2011b). Copyright 2011 Elsevier.

In Equation (7), *m*_*Ee*_is the moles of target that elute at time *t* from the column, and *m*_*Eo*_is the original moles of target that were present on the column. When plotting $\ln\left(\frac{dm_{Ee}\;}{dt}\right)$ vs. *t, k*_*d*_ can be obtained from the negative slope of the resulting plot (Chen et al., 2009; Schiel and Hage, 2009; Bi et al., 2015; Zheng et al., 2015b). Some plots that have been obtained in the manner by the peak decay method are shown in Figure 7 (Yoo and Hage, 2011b).

The peak decay method has been used to investigate several types of biological interactions. This method was originally used in a competitive format to study the dissociation rate of the sugar 4-methylumbelliferyl α-D-mannopyranoside from Con A in the presence of the competing agent 4-methylumbelliferyl α-D-galactopyranoside (Moore and Walters, 1987). The non-competitive peak decay method has been utilized to study the interactions of many drugs with serum proteins. This second approach has been used to determine the dissociation rate constants of imipramine, diazepam, tolbutamide, acetohexamide, quinidine, amitriptyline, verapamil, lidocaine, and nortriptyline with AGP or HSA (Yoo and Hage, 2011a). The binding of *R*-warfarin with monolith columns containing immobilized HSA has also been studied by this method (Yoo and Hage, 2011b). In addition, peak decay analysis has been utilized to determine the dissociation kinetics of immobilized antibodies with 2,4-dichlorophenoxyacetic acid and related herbicides for the selection of elution conditions in immunoaffinity chromatography (Nelson et al., 2010). This approach has further been used to examine the interaction kinetics of immobilized anti-thyroxine antibodies or aptamers with thyroxine, as well as the dissociation of immunoglobulin G (IgG)-class antibodies from protein G columns (Pfaunmiller et al., 2012; Anguizola et al., 2018).

Peak decay analysis has been employed to study many systems exhibiting weak-to-moderate binding interactions (*K*_*a*_ ≤ 10^6^ M^−1^) (Chen et al., 2009; Schiel and Hage, 2009; Nelson et al., 2010; Yoo and Hage, 2011a,b; Bi et al., 2015; Zheng et al., 2015b). However, peak decay is also an effective method for studying the elution conditions that are needed for systems that exhibit much strong binding under their application conditions. Some examples of binding agents that belong to this second category are antibodies, aptamers and protein G (Hage et al., 2012; Pfaunmiller et al., 2012; Anguizola et al., 2018). This set of methods can be applied in cases where the analyte undergoes fast dissociation from the immobilized binding agent, with a *k*_*d*_ in the range of 10^−2^-10^1^ s^−1^ (Chen et al., 2009; Schiel and Hage, 2009; Nelson et al., 2010; Yoo and Hage, 2011a,b; Pfaunmiller et al., 2012; Bi et al., 2015; Zheng et al., 2015b; Anguizola et al., 2018).

Because peak decay analysis is based on linear regression and an elution profile with a logarithmic-based response, data analysis is usually easier to conduct than in band-broadening measurements (Chen et al., 2009; Schiel and Hage, 2009; Yoo and Hage, 2011a,b). This same feature makes peak decay analysis appealing for the study and optimization of elution conditions. If non-specific binding is present in the system, similar studies need to performed using a control column to identify and correct for these effects. Another limitation of this method is that the conditions that are needed to make the target re-association negligible, and dissociation the rate determining step, may be difficult to achieve for some systems (Nelson et al., 2010; Yoo and Hage, 2011a,b; Zheng et al., 2015b).

## Split-Peak Method

**Figure 8:**
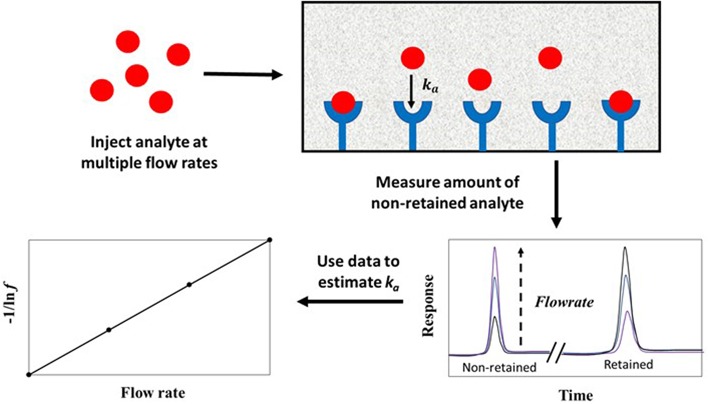
General scheme used for studying analyte interactions with an immobilized binding agent by the split-peak method. Terms: *k*_*a*_, association rate constant; *f*, free fraction of the analyte which is non-retained by the column.

The split-peak method is an approach for studying solute-ligand interactions in an environment where the solute binds irreversibly to an immobilized binding agent. This approach, which is illustrated in Figure 8, is based on the probability (under appropriate application conditions) that a small fraction of an injected analyte may not interact with the stationary phase as the analyte passes through a column. This portion elutes as a non-retained peak; the remainder of the analyte is retained and later elutes from the column. The resulting phenomenon is called the “split-peak effect” (Hage et al., 1986). The split-peak effect increases as the sample residence time in the column decreases or as the injection flow rate increases (Hage et al., 1986; Schiel and Hage, 2009; Bi et al., 2015; Zheng et al., 2015b).

### Methods Using Linear Elution Conditions

The split-peak effect can be employed to obtain the association rate constant (*k*_*a*_) for an injected analyte with an immobilized binding agent under linear elution conditions by using Equation (8) (Hage et al., 1986).

(8) $$\begin{array}{l} -\frac{1}{\text{ln }f}\;=\;F\;\left(\frac{1}{k_{1}V_{e}\;}\;+\;\frac{1}{k_{a}m_{L}\;}\right) \end{array}$$

The term *f* is the free fraction of the analyte (which is non-retained by the column), *F* is the injection flow rate, m_L_ is the moles of active binding agent, and *V*_*e*_ is the interparticle (or excluded) volume of the mobile phase. The term *k*_*a*_ is the association rate constant for the analyte and immobilized binding agent, and *k*_1_ is the forward mass transfer rate constant, which describes the movement of analyte from the flowing mobile phase region to the stagnant mobile phase (Hage et al., 1986).

**Figure 9:**
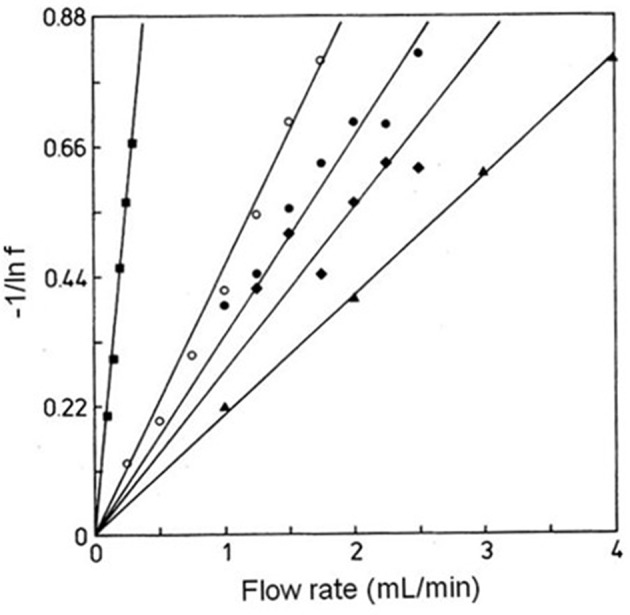
Plots of −1/ln *f* vs. *F* for examining interactions of rabbit IgG with immobilized protein A by the split-peak method. These experiments were conducted using various sample sizes, immobilization methods, and support materials, as represented by the plots in (◦), (•), (▲), (■), and (♦). Terms: *f*, free fraction of the analyte. Reproduced with permission from Hage et al. (1986). Copyright 1986 American Chemical Society.

According to Equation (8), a plot of −1/ln *f* against *F* should result in a linear plot when a small amount of analyte is injected (i.e., linear elution conditions). This equation also indicates that the measured free fraction may be affected by the presence of slow stagnant mobile phase mass transfer, as described by 1/(*k*_1_*V*_*e*_), or slow adsorption to the immobilized binding agent, as represented by 1/(*k*_*a*_*m*_*L*_). If the slow step in analyte retention is adsorption, the slope of a plot of −1/ln *f* vs. *F* can provide *k*_*a*_ if the value of *m*_*L*_ is also known (Hage et al., 1986; Schiel and Hage, 2009; Zheng et al., 2015b). This type of plot is shown in Figure 9 (Hage et al., 1986). If stagnant mobile phase mass transfer is the rate-limiting step, the slope can be used to obtain *k*_1_. Intermediate cases, in which both adsorption and stagnant mobile phase mass transfer are important in determining the rate of binding by the analyte, can also occur (Hage et al., 1986; Hage and Walters, 1988). This technique has been employed to study the association kinetics of IgG-class antibodies with HPAC columns that contain protein G, protein A or a mixture of protein G and protein A (Hage et al., 1986; Rollag and Hage, 1998; Anguizola et al., 2018). This approach has also been utilized to optimize the retention and determination of human IgG in clinical samples by means of HPAC and protein A columns (Hage and Walters, 1987).

### Methods Using Non-linear Conditions

The split-peak method can also be used under non-linear conditions. For instance, the slopes for plots obtained according to Equation (8) can be measured at several known amounts of injected analyte and extrapolated to an infinitely small sample concentration (Hage et al., 1986; Hage and Walters, 1988; Rollag and Hage, 1998). It is further possible to use alternative expressions to Equation (8) that can be used in special cases where the amount of analyte is not negligible. Equation (9) shows one such relationship for a situation in which the rate-limiting step in retention is adsorption of the analyte to the immobilized binding agent (Hage et al., 1993; Vidal-Madjar et al., 1997).

(9) $$\begin{array}{l} f\;=\;\frac{S_{o}\;}{Load\;A}\text{ ln}\left[1\;+\left(e^{Load\;A/S_{o}}-1\right)\;{\;e}^{-1/{\;S}_{o}\;}\right] \end{array}$$

In this equation, *Load A* is the relative amount (mol/mol binding agent) of the analyte that is applied to the column. The term *So* is a value equal to *F/k*_*a*_*m*_*L*_, in which each of the individual parameters are the same as described for Equation (8). This modified split-peak method has been employed in a number of studies to measure the association rate constants of immobilized anti-HSA antibodies with HSA in various assay and injection formats (Hage et al., 1993, 1999; Vidal-Madjar et al., 1997). This expression and approach have also been utilized to determine the association rate constants of herbicides and thyroxine with antibodies in HPAC columns (Nelson et al., 2010; Pfaunmiller et al., 2012).

The split-peak method has been employed with both linear and non-linear conditions to study a variety of systems that exhibit high binding affinities (*K*_*a*_ > 10^6^ M^−1^). Association rate constants determined by this method have typically been in the range of 10^4^-10^6^ M^−1^ s^−1^. One advantage of this approach is that it uses peak area measurements, which are often easier to acquire and more precisely analyzed than peak variances (Hage et al., 1986; Vidal-Madjar et al., 1997; Rollag and Hage, 1998; Nelson et al., 2010; Pfaunmiller et al., 2012). One limitation is this method is best suited for biological reactions that have slow dissociation (i.e., *k*_*d*_ < 10^−1^ s^−1^) and that allow a good separation between the retained and non-retained fractions of an analyte (Nelson et al., 2010; Pfaunmiller et al., 2012). Furthermore, the column size, flow rate, and other experimental conditions need to be selected in such a way that the split-peak effect is observable (Schiel and Hage, 2009; Zheng et al., 2015b).

## Peak Fitting

Peak fitting is based on the application of a relatively large amount of a target analyte to an affinity column and fitting the peaks that are observed at known sample concentrations or conditions to a given chromatographic model. The best-fit parameters for the peak are utilized to get the equilibrium and rate constants for the target's interaction with the binding agent (Moaddel and Wainer, 2007; Moaddel et al., 2007). Peak fitting can be performed using either zonal elution or frontal analysis as the format by which the analyte is applied to the column (Schiel and Hage, 2009; Bi et al., 2015; Zheng et al., 2015b).

### Methods Using Zonal Elution

**Figure 10:**
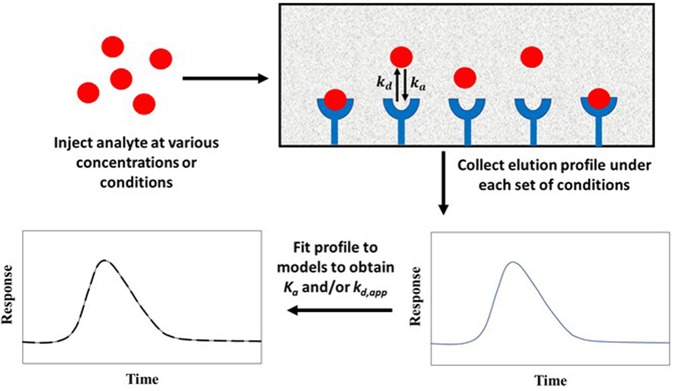
General scheme for studying analyte interactions with an immobilized binding agent by peak fitting. Terms: *K*_*a*_, association equilibrium constant; *k*_*a*_, association rate constant; *k*_*d*_, dissociation rate constant; *k*_*d,app*_, apparent dissociation rate constant.

Figure 10 shows a typical scheme for carrying out peak fitting under zonal elution conditions. The results of such an experiment can be analyzed by employing Equation (10) (Wade et al., 1987; Moaddel and Wainer, 2007; Moaddel et al., 2007).

(10) $$\begin{array}{l} y\;=\;\frac{a_{o}\;}{a_{3}\;}\;\left[1-e^{\left(\frac{-a_{3}\;}{a_{2}\;}\right)}\right]\left[\frac{\left(\sqrt{\frac{a_{1}\;}{x}\;}\right)\;I_{1\;}\left(\frac{2\;\sqrt{a_{1}x\;}}{a_{2}\;}\right)\;e^{-x-a_{1}\;/a_{2}\;}\;}{1-T\;\left(\frac{a_{1}\;}{a_{2}\;},\;\frac{x}{a_{2}\;}\right)\left[1-e^{-a_{3}\;/a_{2}\;}\right]}\right] \end{array}$$

The value of *y* in this equation is the measured signal at a given reduced retention time *x*. The term *I*_1_ is a modified Bessel function, and *T* is the switching function. The factors *a*_*o*_, *a*_1_, *a*_2_, and *a*_3_ are the parameters obtained by fitting experimental data to Equation (10). These parameters can be used to determine the equilibrium constant and rate constants for the interaction of the analyte with the immobilized agent. For instance, the association equilibrium constant can be found by using the relationship *K*_*a*_ = *a*_3_*/C*_*o*_, and the apparent dissociation rate constant can be obtained by using *k*_*d,app*_ = *1/a*_2_*t*_*M*_, where *t*_*M*_ is the column void time and *C*_*o*_ is related to the analyte's concentration, the sample volume, and the column's dead volume (Moaddel and Wainer, 2007; Schiel and Hage, 2009).

This method was first employed to characterize the interaction kinetics between *p*-nitrophenyl-α-D-mannopyranoside and immobilized Con A (Wade et al., 1987). This equation has also been utilized to study the binding, rate constants, and quantitative-structure activity relationships for interactions of nicotinic acetylcholine receptor (nAChR) with phencyclidine, 18-methoxycoronaridine, and bupropion plus verapamil (Jozwiak et al., 2002, 2003, 2004, 2007; Moaddel et al., 2005, 2007). Peak fitting has been utilized to measure rate constants for the interactions of novobiocin with heat shock protein 90α (Marszałł et al., 2008). This method has also been applied to investigate the rate constants and types of binding sites for drug interactions with β_2_-AR (Li et al., 2015; Liang et al., 2018) and to examine drug binding with β-cyclodextrin with detection based on mass spectrometry (Wang et al., 2016). Similar peak fitting approaches have been described for kinetic studies of IgG with immobilized protein A during elution at pH 3.0 and to determine rate constants for the binding of lysozyme with immobilized Cibacron Blue 3GA at various concentrations of sodium chloride (Lee and Chuang, 1996; Lee and Chen, 2001).

### Methods Using Frontal Analysis

Another way peak fitting can be used for studying biological interactions is with frontal analysis. This combination involves continuously applying an analyte solution to the column. Equation (11) shows one way an association rate constant (*k*_*a*_) can be determined by measuring an apparent association rate constant (*k*_*a, app*_) with frontal analysis (Renard et al., 1995).

(11) $$\begin{array}{l} \frac{1}{k_{a,app}}\;=\;\frac{q_{x\;}V_{M}\;}{F\;n_{mt}}\;+\;\frac{1}{k_{a}} \end{array}$$

In this equation, *n*_*mt*_ represents the global mass transfer coefficient (i.e., a term dependent on the support size and dimensions of the column), *F* is the flow rate, *V*_*M*_ is the void volume, and *q*_*x*_ is the loading capacity of the column per unit volume of mobile phase. This equation assumes analyte dissociation from the immobilized agent is not significant during the time of the experiment (Renard et al., 1995; Schiel and Hage, 2009; Zheng et al., 2015b). If this assumption is true, a linear relationship should be produced by a plot of 1/*k*_*a,app*_ vs. *q*_*x*_. The association rate constant *k*_*a*_ can be determined from the intercept of this plot (Renard et al., 1995). This approach makes it possible to correct for the band-broadening contributions to *k*_*a, app*_ due to stagnant mobile phase mass transfer, and has been utilized to find the association rate constant of anti-HSA antibodies with HSA (Renard et al., 1995).

A second way peak fitting can be performed with frontal analysis is by utilizing Equation (12) (Munro et al., 1993, 1994; Schiel and Hage, 2009).

(12) $$k_{d\;}=\;\frac{2(V_{A}-V_{A}{}^{*})}{d\sigma_{A}{\;}^{2}\;/dF}$$

In this equation, *V*_*A*_ and $V_{A}^{*}$ are the breakthrough volumes for the retained analyte and a non-retained solute. The term *k*_*d*_ is the dissociation rate constant, *F* is the flow rate, and ${\sigma_{A}\;}^{2}$ is the variance of the breakthrough curve for the analyte. When a plot of ${\sigma_{A}}^{2}$ vs. *F* is made, the slope $\left(d\frac{{\sigma_{A}\;}^{2}}{dF}\right)$ that is obtained can be used with the other terms in Equation (12) to calculate *k*_*d*_. One advantage of this method is it can be conducted using various sample concentrations to provide a set of values for $\left(d\frac{{\sigma_{A}}^{2}}{dF}\right)$ to estimate *k*_*d*_ (Munro et al., 1993, 1994; Schiel and Hage, 2009; Zheng et al., 2015b). This technique has been employed in measuring the dissociation rate constant that describes the interaction of *p*-nitrophenyl-α-D-mannopyranoside with Con A (Munro et al., 1993, 1994).

An advantage of peak fitting is it can be used to study systems exhibiting weak-to-moderate binding under non-linear conditions (Schiel and Hage, 2009; Zheng et al., 2015b). The range of association and dissociation rate constants that have been reported when using this method have spanned from 10^4^-10^7^ M^−1^ s^−1^ to 10^−1^-10 s^−1^, respectively (Moaddel and Wainer, 2007; Moaddel et al., 2007; Schiel and Hage, 2009; Zheng et al., 2015b). One limitation of peak fitting methods is it is necessary to test and verify any assumptions that are made. For instance, it may be necessary to determine whether mobile phase mass transfer effects need to be considered (Renard et al., 1995; Schiel and Hage, 2009; Zheng et al., 2015b).

## Ultrafast Affinity Extraction

**Figure 11:**
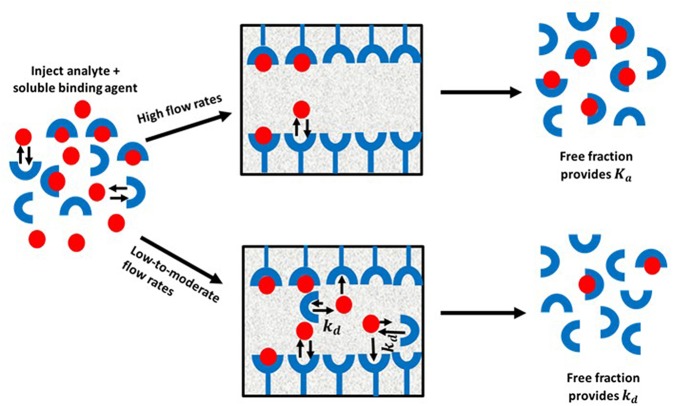
General scheme for studying analyte interactions with a soluble binding agent by using ultrafast affinity extraction. Terms: *K*_*a*_, association equilibrium constant; *k*_*d*_, dissociation rate constant.

Ultrafast affinity extraction is a yet another tool that can be used with affinity chromatography and HPAC for kinetic studies. Figure 11 illustrates the basic principle of this approach. This method can be used to measure the free or non-bound fraction of an analyte in a mixture of the analyte and a soluble form of the binding agent. This approach was initially developed to measure equilibrium constants for drug-protein binding, as can be obtained from free fractions measured at high flow rates (Mallik et al., 2010; Zheng et al., 2013). However, a modified form of this method has also been reported that can measure both the thermodynamics and kinetics of solute-ligand interactions in solution by using intermediate and high flow rates (Zheng et al., 2014b).

When ultrafast affinity extraction is utilized for kinetic studies, a sample containing a mixture of the analyte and soluble binding agent of interest is injected onto a small HPAC column. If this injection is made at a sufficiently high flow rate, the short residence time of the sample mixture in the column will avoid or minimize release of the analyte from its complex with the soluble binding agent. This causes a separation of the bound and free fractions of the analyte as the analyte's free form is quickly extracted by the immobilized binding agent. These conditions make it possible to obtain the association equilibrium constant (*K*_*a*_) of the analyte with the soluble binding agent through the measured free fraction and the known total concentrations of the analyte and binding agent that were present in the sample. On the other hand, injection at low-to-moderate flow rates will result in longer residence times in the column for the sample and increase the chance of the analyte dissociating from the soluble binding agent. This dissociation will increase the apparent free fraction that is measured for the analyte and will provide information on the interaction kinetics of the analyte with binding agent(s) in the sample (Zheng et al., 2014b, 2015a).

There are two equivalent relationships that can be used to obtain dissociation rate constants when employing ultrafast affinity extraction. These relationships are given in Equations (13) and (14) (Zheng et al., 2014b, 2015a).

(13) $$\begin{array}{l} \ln\frac{1\;-f_{o}\;}{1\;-f_{t}\;}=k_{d}\text{t} \end{array}$$

(14) $$\begin{array}{l} \ln\frac{1\;}{1\;-f_{t}\;}=k_{d}\text{t}-ln\left(1-f_{o}\right) \end{array}$$

**Figure 12:**
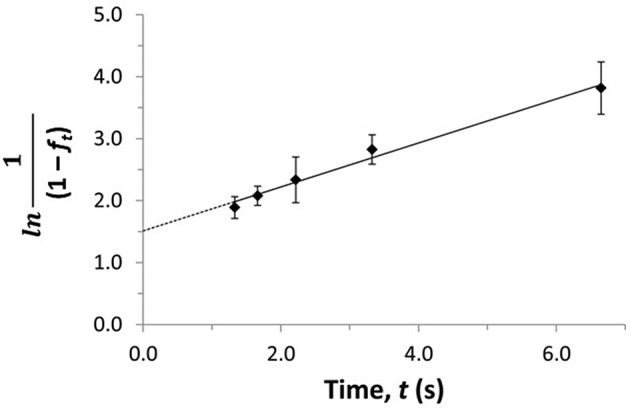
Plot of ln[1/(1–*f*_*t*_)] vs. *t* to estimate the dissociation rate constant for verapamil with soluble HSA by using ultrafast affinity extraction and Equation (14). Terms: *f*_*t*_, apparent free fraction for the analyte measured at a given dissociation time; *t*, dissociation time. Adapted with permission from Zheng et al. (2014b). Copyright 2014 American Chemical Society.

The term *f*_*o*_ represents the free or non-bound fraction of A that is present in a mixture of A and the soluble binding agent at equilibrium (i.e., as measured at high flow rates), while *f*_*t*_ is the apparent free fraction for A that is observed in the presence of dissociation of the analyte-binding agent complex in the sample (i.e., as measured at lower flow rates). The term *t* is the time this dissociation is allowed to occur, as given by the residence time of the sample in the column. If the dissociation process follows pseudo-first order decay, Equations (13) and (14) predict that a linear relation should be obtained when plotting ln[(1-*f*_*o*_)/(1-*f*_*t*_)] or ln[1/(1-*f*_*t*_)] against *t*. The slope of these plots can be used to obtain *k*_*d*_ (Zheng et al., 2014b, 2015a). Figure 12 shows a plot that has been prepared in this manner with Equation (14) and used to determine the value of *k*_*d*_ by means of ultrafast affinity extraction (Zheng et al., 2014b).

Ultrafast affinity extraction has been used as a tool to study the kinetics of many drugs with transport proteins. For instance, this method has been used to examine dissociation rate constants of HSA with warfarin, tolbutamide, acetohexamide, verapamil, gliclazide, chlorpromazine, diazepam, tolbutamide, quinidine, glimepiride, glibenclamide, and glipizide (Zheng et al., 2014b, 2016; Beeram et al., 2018; Yang et al., 2018). Similar work with AGP has been conducted to examine the dissociation rates of verapamil, lidocaine, imipramine, disopyramide, chlorpromazine and propranolol (Beeram et al., 2017, 2018). The same approach has been utilized to determine dissociation rate constants of testosterone with HSA, sex hormone binding globulin (SHBG), and equine serum albumin (ESA) (Zheng et al., 2015a; Czub et al., 2019).

The ultrafast affinity extraction method has been used to study systems that have weak-to-strong affinities (10^3^-10^9^ M^−1^). The dissociation rate constants obtained by this technique have been in the general range of 10^−2^-10 s^−1^ (Zheng et al., 2014b, 2015a, 2016; Bi et al., 2015; Beeram et al., 2017, 2018; Yang et al., 2018). One advantage of this method is it makes use of moderate-to-high flow rates, which can result in analysis times of only a few minutes. This method also uses only small volumes of the injected sample (i.e., a few μL) and can directly measure the dissociation rate constants for a solute and binding agent in solution. Another advantage is this method uses peak areas instead of peak fitting or band-broadening measurements, which can result in precise estimates of rate constants. One potential limitation of this method is that the column sizes and flow rates that are needed to separate the free and bound forms of the analyte currently need to be optimized for each solute-binding agent system (Zheng et al., 2014b, 2015a; Bi et al., 2015; Beeram et al., 2018). However, previous work has provided a number of guidelines that can be utilized to aid in this process (Beeram et al., 2018).

## Conclusions

This review discussed various techniques which can be employed in affinity chromatography or HPAC for studying the rates of solute interactions with immobilized or soluble binding agents. These techniques included those based on plate height measurements, peak profiling, peak decay analysis, the split-peak effect, peak fitting, and ultrafast affinity extraction. The association rate constants and dissociation rate constants that have been determined by these methods have spanned from 10^3^-10^7^ M^−1^ s^−1^ to 10^−2^-10 s^−1^, and represent systems with weak-to-strong binding constants ranging from 10^3^ to 10^9^ M^−1^ (Schiel and Hage, 2009; Bi et al., 2015; Zheng et al., 2015b).

These methods have several common advantages. For instance, the affinity column and immobilized binding agent can often be reused for many experiments (i.e., up hundreds of studies), ensuring that good reproducibility and precision are obtained for the kinetic measurements. In addition, many of these methods are “label-free” and can be used with standard HPLC systems, which allows them to be automated and used with a variety of detectors. Detection methods used with HPAC or affinity chromatography in these applications have included absorbance, fluorescence, and mass spectrometry (Schiel and Hage, 2009; Bi et al., 2015; Zheng et al., 2015b). One possible limitation for many of these methods is immobilization of the binding agent is needed; this means that conditions for immobilization should be selected and validated to ensure the solute's interaction with the binding agent is similar to what would be seen in their natural environment (Schiel and Hage, 2009; Bi et al., 2015; Zheng et al., 2015b). One route to overcome this issue is to use the affinity column to indirectly study binding by a target compound with a soluble binding agent, as occurs in ultrafast affinity extraction (Bi et al., 2015; Zheng et al., 2015b).

The applications of these methods have included measurements for the rate constants of drugs as they interact with serum proteins, the binding of antibodies with antigens, the interactions of receptors with inhibitors, and the binding sugars or sugar analogs with lectins. The information that has been obtained on these interactions is of great importance in fields such as clinical chemistry, pharmaceutical science and biomedical research. Based on the current work in this field, and the advantages of using affinity chromatography and HPAC to obtain this information, even more applications are expected as further improvements and advances occur with these methods. Examples of some potential applications include high-throughput screening of drug candidates, use of chromatographic kinetic studies in multi-dimensional systems, and combined use of these methods with other techniques to provide both functional and structural data in areas such as proteomics, glycomics and personalized medicine (Anguizola et al., 2013; Zheng et al., 2013, 2014a; Matsuda et al., 2014).

## Author Contributions

SI and DH conducted the literature research that lead to this review and were responsible for proofing and preparing the final copy of this review. SI prepared the initial draft with input from DH and a section from SO on the topic of peak fitting.

### Conflict of Interest

The authors declare that this manuscript was prepared, and the associated work described in this paper by the authors was conducted, in the absence of any commercial or financial relationships that could be construed as a potential conflict of interest.
